# Supporting teachers and children in schools: the effectiveness and cost-effectiveness of the incredible years teacher classroom management programme in primary school children: a cluster randomised controlled trial, with parallel economic and process evaluations

**DOI:** 10.1186/1471-2458-12-719

**Published:** 2012-08-30

**Authors:** Tamsin Ford, Vanessa Edwards, Siobhan Sharkey, Obioha C Ukoumunne, Sarah Byford, Brahm Norwich, Stuart Logan

**Affiliations:** 1 Child Mental Health Research Group, University of Exeter Medical School, Vesey Building, Salmon Pool Lane, Exeter, EX2 4SG, UK; 2 Collaboration for Leadership in Applied Health Research and Care for the South West Peninsula, University of Exeter Medical School, Veysey Building, Salmon Pool Lane, Exeter, EX2 4SG, UK; 3 Centre for the Economics of Mental and Physical Health, Institute of Psychiatry, Kings College London, De Crespigny Park, London, SE5 8AF, UK; 4 Graduate School of Education, University of Exeter, St Luke’s Campus, Exeter, EX1 2LU, UK

**Keywords:** Antisocial behaviour, Socio-emotional regulation, Mental health, Children, Cost-effectiveness

## Abstract

**Background:**

Childhood antisocial behaviour has high immediate and long-term costs for society and the individual, particularly in relation to mental health and behaviours that jeopardise health. Managing challenging behaviour is a commonly reported source of stress and burn out among teachers, ultimately resulting in a substantial number leaving the profession. Interventions to improve parenting do not transfer easily to classroom-based problems and the most vulnerable parents may not be easily able to access them. Honing teachers’ skills in proactive behaviour management and the promotion of socio-emotional regulation, therefore, has the potential to improve both child and teacher mental health and well-being and the advantage that it might potentially benefit all the children subsequently taught by any teacher that accesses the training.

**Methods/Design:**

Cluster randomised controlled trial (RCT) of the Incredible Years teacher classroom management (TCM) course with combined economic and process evaluations.

One teacher of children aged 4–9 years, from 80 schools in the South West Peninsula will be randomised to attend the TCM (intervention arm) or to “teach as normal” (control arm). The primary outcome measure will be the total difficulties score from the Strengths and Difficulties Questionnaire (SDQ) completed by the current class teachers prior to randomisation, and at 9, 18 and 30 months follow-up, supplemented by parent SDQs. Secondary measures include academic attainment (teacher report supplemented by direct measurement in a sub-sample), children’s enjoyment of school, and teacher reports of their professional self-efficacy, and levels of burn out and stress, supplemented by structured observations of teachers classroom management skills in a subsample. Cost data for the economic evaluation will be based on parental reports of services accessed. Cost-effectiveness, using the SDQ as the measure of effect, will be examined over the period of the RCT and over the longer term using decision analytic modelling. The process evaluation will use quantitative and qualitative approaches to assess fidelity to model, as well as explore Head teacher and teachers’ experiences of TCM and investigate school factors that influence the translation of skills learnt to practice.

**Discussion:**

This study will provide important information about whether the Teacher Classroom Management course influences child and teacher mental health and well-being in both the short and long term. It will also provide valuable insights into factors that may facilitate or impede any impact.

The trial has been registered with ISCTRN (Controlled Trials Ltd) and assigned an ISRCTN number ISRCTN84130388. (http://www.controlled-trials.com/isrctn/search.html?srch=ISRCTN84130388&sort=3&dir=desc&max=10)

## Background

### Existing research

Childhood psychopathology is common and the prevalence of antisocial behaviour in particular has increased in recent years [1,2]. Poor socio-emotional adjustment in early childhood increases the risks of psychiatric disorder, risk taking behaviour, educational failure, and involvement in crime in both childhood and adulthood [1,2]. Affected children incur substantial costs to both society and their families [3]. The impairment and societal costs of antisocial behaviour, however, are evident throughout the population distribution rather than just among those with the highest level of problems [3]. Children in poorly managed classrooms observe that disruptive behaviour commands staff attention while good behaviour is rarely acknowledged, which may contribute to later disruptive behaviour and / or disaffection and disengagement from secondary school with the attendant risks to health and educational attainment. Teachers often complain of lack of training to manage disruptive behaviour, which is associated with higher stress levels and burn out [4-6]. An intervention that supports teachers to manage disruptive behaviour and promote socio-emotional competence could potentially benefit every child subsequently taught by that teacher as well as the teacher themselves, and could therefore be substantially more cost-effective than direct intervention with subsequent cohorts of children.

Despite a multitude of programmes targeting children [7], a recent systematic review identified that only two interventions focusing on enhancing teachers’ skills that had been studied more than once; [8] one of these was the Incredible Years (IY) Teacher Classroom Management (TCM) course. TCM was also the programme with the most robust evidence, despite the fact that there have been only two trials of TCM in isolation from other interventions [9,10]. In a small observational study in Wales, 23 teachers reported high levels of satisfaction with the TCM course. Direct observation revealed that teachers who had accessed TCM gave clearer instructions, allowed more time for compliance and their pupils were more compliant [9]. A subsequent RCT involved 12 classes (16 teachers) from 11 primary schools and 107 children aged 4–5 years [9]. The results indicated weak evidence that TCM produced changes in teachers’ behaviour and their pupil’s behaviour [9]. A second trial in Limerick involved 11 schools, 22 teachers and 207 children aged 4–7 years and detected changes in teacher’s behaviour on both observation and self-report (. There was little evidence of change in teacher-rated child well-being [10]. They estimated the intervention to cost approximately €2000 per teacher or €100 per child in schools with a class size of 20. Neither trial has been published in peer reviewed journals. Follow-up into a second year in Wales has been completed but results are not yet available, while follow-up in the Irish study is ongoing. Both studies suggest that TCM is potentially sufficiently intense to change teachers’ behaviour. Other studies involving TCM have either added additional coaching for teachers or children, and/or studied the parallel parent and child programmes with or without TCM. All suggest that TCM is potentially effective but given the additional interventions, it is impossible to estimate the impact of TCM alone as a public health intervention [11-14]. We are unaware of any other planned or ongoing trials of TCM in isolation from other interventions.

### The intervention: the teacher classroom management programme

The intervention being evaluated is the IY Teacher Classroom Management (TCM) programme [14]. TCM draws on cognitive social learning theory, particularly Patterson’s theories [15] about how coercive cycles of interaction between adults and children reinforce unwanted behaviour patterns, Bandura’s ideas [16] about the importance of modelling and self-efficacy, and Piaget’s developmental interactive learning methods [17]. In addition, it also incorporates strategies for challenging angry, negative, and depressive internal dialogue in adults whilst interacting with children, drawn from cognitive behavioural approaches. TCM is delivered to groups of ten teachers, and involves six whole-day sessions spread over a period of six months. It is delivered in a collaborative style with group leaders encouraging all to share their experience and expertise and to value that of others. The explicit goals are to: a) enhance teacher classroom management skills and to improve teacher-student relationships; b) assist teachers to develop effective group and individual behaviour plans to enable proactive rather than reactive classroom management; c) encourage teachers to adopt and promote social and emotional regulation skills; and d) encourage teachers to strengthen positive teacher-parent relationships with all parents.

TCM uses goal setting, reflective learning, video modelling, role play, rehearsal of novel management strategies, group discussion, support and problem solving, and cognitive and emotional self-regulation training. Teachers are encouraged to experiment with novel strategies between sessions and to discuss their resulting experiences. The intervention will be delivered by suitably trained and supervised ‘*group leaders’*. We envisage the same process of delivery should the trial support the implementation of TCM. The Incredible Years (IY) Foundation is collaborating to ensure that our group leaders deliver the intervention to the highest possible level and with fidelity to model. The comparator is teaching as usual (TAU). Plymouth, Torbay and Devon Local Authorities have a range of behavioural support and educational psychology services, and teachers in both arms will be able to access whatever other resources are available to them. Precisely what is accessed and how TCM might complement or supplement what is already available will be a major focus of the qualitative aspect of the process evaluation.

## Need for the current study

This study is necessary despite previous evaluations of the IY programmes, as it will be only the third evaluation of TCM in isolation from other interventions, and will be the only one sufficiently powered to detect an effect on children’s well-being. In addition, we are extending the upper age limit of children studied from seven to nine years, widening the outcome measures to include academic attainment and child and parent ratings of child well-being, focusing on the potential for particularly high impact on children living in deprived circumstances and conducting an evaluation of cost-effectiveness, none of which were a feature of earlier studies. TCM has the potential to make a major impact on the current and future mental health and well-being of the school-age population of England and to lead to substantial public sector cost savings in both the short and longer term. This study will also examine the influence of deprivation at both the school and individual level by the use of individual family and school post code to link to the Index of Multiple Deprivation and individual and school level data on free school meals. Young boys from socio-economically disadvantaged areas and / or families are particularly vulnerable to developing impairing levels of anti-social behaviour and it is important to study the impact of any intervention on this high risk group.

## Methods/Design

### Aims/objectives

· To evaluate whether TCM improves socio-emotional well-being among children as measured by the teacher completed Strengths and Difficulties Questionnaire (SDQ) cross-validated with direct observation, parental SDQ and child report on How I Feel About My School where available.

· To evaluate whether TCM improves academic attainment as measured by teacher assessment of pupil progress (APP) cross-validated with standardised assessments and SATS where available.

· To evaluate whether any improvements in well-being and attainment are sustained over the next two academic years.

· To evaluate whether TCM reduces ‘burn out’ and improves self-efficacy and well-being among teachers using the Maslach Burn Out Inventory, the Teacher Self-Efficacy Questionnaire and the Everyday Feelings Questionnaire.

· To evaluate whether TCM improves teacher’s classroom management skills using the behavioural management strategies reported by teachers in the Teacher Satisfaction Questionnaire.

· To use qualitative methods to investigate how teachers apply the strategies suggested by TCM in the classroom and any factors that may influence this process, including: year group taught, school climate and additional support and advice available to them.

· To evaluate both the utility of TCM to teachers in their practice one year after attending the course and how TCM is related to additional sources of behavioural support and school context.

· To evaluate the cost and cost-effectiveness of TCM compared to teaching as usual at final follow-up.

· To extrapolate the results from the randomised controlled trial (RCT) into young adulthood using decision analytic modelling and published data to explore the longer-term cost and cost-effectiveness implications of TCM compared to teaching as usual and to model potential cost savings in the longer term.

### Setting

The setting is primary schools within Devon, Torbay and Plymouth.

#### *Inclusion criteria*

· Teachers, parents and children in primary, state run, mainstream schools in Devon, Torbay or Plymouth with at least one single year group class of 15 or more pupils in Reception or Years 1–4. This will provide a sample of children aged 4–9 years at recruitment.

· To be eligible, the nominated teacher must have classroom responsibility for a single year group class for a minimum of four days per week.

### Exclusion criteria

· Schools that have only mixed year group classes, all classes have fewer than 15 children, are under “special measures”, are privately funded or are without a substantive head teacher.

· Teachers on contracts of less than three years.

· Children with so little use of spoken English that they are unable to complete the measures, even with support.

· Children whose parent(s) do not have a sufficient use of English to enable them to give consent for their child to participate or answer questionnaires, even with assistance.

Recruiting teachers and children from classes containing single year groups is vital to preserve the original allocation group status for follow up. Provided that we start with single year groups, the original control group children will then be with a new teacher in subsequent academic years allowing the control teachers to access TCM. It does not affect the trial design for children to graduate into mixed year group classes after the first year of participation, so schools only need to have one single year group class to be eligible to participate.

### Design

#### *Cluster randomised controlled trial*

The core of the study will be a pragmatic cluster randomised controlled trial with one teacher and their pupils per primary school (cluster) allocated to TCM training or teaching as usual (TAU). The recruitment plan is dictated by the school academic year and the duration of the intervention (six months), which means that the TCM course can only run once per academic year. Eighty schools will be recruited over three year; 15 in Cohort 1, 30 in Cohort 2 and 35 in Cohort 3.

Each school will participate in the trial for three academic years. Child and teacher outcomes will be assessed at the beginning and end of the first academic year (T0 and T1). At the end of the first academic year, teachers and children will separate; the study children will have new teachers in each follow up year (T2 and T3), who will complete the child well-being measures even if a child moves school. The study teachers will also be working with a new class of children, which allows us to offer the control teachers TCM in the second year of participation (i.e. *after* recruitment) as an incentive for participation. Child outcomes will be measured in both follow-up years (T2 and T3) as part of the follow up trial design. Schools sometimes move teachers around year groups for school improvement purposes, so the situation may arise when a teacher and children participating in the trial may come back into contact with each other during the follow up period. Control teachers will be offered access to TCM in the second year of participation in the trial, so contact between control children and control teachers would serve to reduce any differences between the arms of the trial, while contact in the second and third years of participation between TCM children and TCM teachers may increase the differences between arms by effectively giving these children a double dose of the intervention. We will explain verbally and in writing to head teachers at recruitment why it is important to avoid a reunion between the teacher and their baseline class during follow-up and we will monitor schools closely to record if this does happen. To prevent contamination and to reflect how the intervention might be rolled out in ‘real life’, the unit of allocation is the school; one teacher per school will participate.

#### *Embedded teacher cohort*

Control teachers will have accessed the course by T2 and T3. As control teachers access the TCM course in the second year of participation, data relating to the teachers (self efficacy, burn out and mental health and well-being) will contribute to the trial outcomes at T1 only when there is a control group. We plan to combine data from teachers in the trial with the same data collected already from the four TCM groups that we have run as part of our Feasibility Studies. Consequently, we will be able to create an uncontrolled cohort of approximately 120 teachers (eight trial groups plus four Feasibility Study groups, each of 10 teachers) to provide additional data using their baseline and national norms for comparison. It is planned that teacher self-report outcomes will be recorded once a year for up to ten years using web-based data collection. This additional study will be the subject of an alternative funding bid.

#### *Economic evaluation*

The economic evaluation will take a broad public sector perspective, including the use of all health, education and social care services, plus criminal justice sector resources and criminal activity. Data will be presented by sector to allow alternative perspectives to be considered separately. Economic data will be collected at T0, T1, T2 and T3; at baseline, information will cover the previous 6 months; at follow-up service use since the previous time-point will be recorded. The cost and cost-effectiveness of TCM compared to TAU will be analysed at the final follow-up point (T3) in terms of the child’s socio-emotional well-being.

#### *Process evaluation*

The process evaluation will combine quantitative and qualitative methodologies, and data collection and analysis will run parallel to the pilot and main phases. Quantitative data relating to the administration and delivery of the TCM course will be collected to record how many sessions were attended by each teacher and to reflect course experience and the adoption of strategies. These will use routine methods of capturing data developed by the Incredible Years Team. Qualitative data will be collected to inform the trial processes and to assess translation of TCM strategies into practice and any impact of TCM on the use of services within schools.

### Data collection

The data for the RCT and economic evaluation will be captured at specified time points. Process evaluation data will be collected at different times in parallel to the main study. Data collection will be via questionnaires (self-report and proxy) completed by teachers, children and parents; classroom observation by independent researchers; academic assessment by teachers and researchers; focus groups/telephone interviews with teachers, headteachers and Special Educational Needs Coordinators (SENCos), and telephone interviews with sub-sets of parents. Additional interviews with parents and children will be subject to a further funding bid if sufficient parents are willing to participate. Teachers will use a web-based electronic data capture system to complete all questionnaire measures on themselves and the children.

### Randomisation and concealment

Schools will be randomised to intervention (TCM course) or control (Teaching As Usual, TAU) through a password protected trial website that will be set up and maintained by the UKCRC accredited Peninsula Clinical Trials Unit. Randomisation will be stratified by the following: *level ofdeprivation atschool level* (below or above 19% of pupil’s eligible for free school meals); *city/non citylocation* (Plymouth/Exeter/Torbay versus other addresses); and *school year* (Key Stage 1 or Foundation and Years 1–2 versus Key Stage 2 or Years 3 and 4). To ensure concealment, all schools within each cohort of recruitment will be randomised simultaneously after the baseline measures have been completed.

We will be unable to blind the staff in schools as to which group they are allocated to. Researchers undertaking the observations of teachers will be external to the core research team and will be kept blind to group allocation at all times, although it is possible that teachers may inadvertently disclose this information to them. We will ask these researchers to guess which groups the teachers that they observed were in at the end of their follow up observations to check if blinding was maintained and to tell us if a teacher informed them about their allocation. Baseline measures will be completed before randomisation and therefore all parties will be blind to allocation at this point. Parents and children are unlikely to be aware of whether their child’s teacher has completed the TCM course and the follow-up measures, with the exception of the service-related interviews completed by parents on a sub-sample and the child measures, are questionnaires that are completed independently and thus difficult for the core team of researchers to influence should they become aware in their liaisons with school about allocation. In addition, the teacher-completed follow-up measures in the second and third year of each school’s participation in the study (T2 and T3) will be completed by a teacher that did not access the intervention, although they are likely to know whether their colleague did or not.

### Assessment and follow up

The trial will start two weeks into the new school year in September. Parents will have two weeks to opt themselves and their child out of the trial. Baseline assessments (T0) will be completed by the October half term holiday. The measures will be completed by the child (on themselves), their parent (on themselves, their child and their child’s use of services) and the class teacher (on the children and themselves). Direct observations in the classroom will take place with a subsample during this time. Following randomisation, the intervention group teachers will then start the TCM course in early November. Literacy/numeracy assessments with a subsample of children will take place in the February/March.

The first follow-up assessment, identical to T0, will be completed in May/June of that first academic year (T1 or nine months post baseline), with the classroom observations completed by researchers blind to allocation.

Each school participates in the trial for three academic years. Only the measures relating to the children (including SDQs completed by their subsequent teachers in the two follow up years) contribute to trial follow-up data at T2 (18 months post baseline) and T3 (30 months post baseline). The teachers will complete the measures relating to themselves to assess the impact of TCM on professional functioning in the longer term, but at T2 and T3 these will no longer contribute to trial outcome data as described earlier. As the recruitment is rolled out over three years, the assessment of different cohorts will be carried out simultaneously within the same school year.

### Trial outcome measures

The primary outcome is child well-being and mental health measured by the teacher-completed Strengths and Difficulties Questionnaire, but this will be supplemented with a number of other measures of child mental health and behaviour. The secondary outcomes are: child attainment and teachers stress, burn out and professional self-efficacy.

#### *Teacher completed measures on each child*

##### Strengths and difficulties questionnaire (SDQ [18])

The teacher-rated version of the SDQ is the *primary outcomemeasure*. The SDQ is a brief, valid and reliable measure of socio-emotional competence that is widely used to assess mental health in childhood. It will be completed by teachers and by parents at all four time-points. The subscales, *behaviour, emotions,overactivity/concentration, peerrelationships andprosocial behaviour,* will allow the examination of particular aspects of well-being in isolation. Responses to the first four add to give a *Total Difficulties* score. Ratings of child distress and impact of difficulties on home life, friendships, classroom learning, and leisure activities combine to form the *Impact Scale*. Teacher SDQs will be cross-validated with parental SDQs, direct observation and the child view of school measure described below.

##### Assessments of pupil progress (APP [19])

APP will be used as the measure of child academic attainment. The APP is completed by all teachers routinely in accordance with detailed guidelines related to the National Curriculum [19] and is a structured approach to periodically assessing children’s level of attainment in mathematics, science, reading, writing and speaking and listening. It enables teachers to track pupils' progress from Year 1 through to the end of year 6. Levels range from eight P levels (working towards Level 1) to Level 5 (above average expectation for a child at the end of year 6); levels 1–5 have three sub-levels (a-c). Using the APP allows us to gather data on academic attainment on all participating children without additional work for teachers and researchers, while the APP approach has proven to be robust, manageable and reliable in practice [19]. APP scores will be supplemented by SATs, which are scored using the same classification as APP, where SATS results are available. Both will result in ordinal data and children are expected to make two points difference on the 12 points of the scale that the age-group under study will reach. Reliability will be further assessed using detailed psychometric tests (WIAT II [20]) in a subsample (see below).

##### Adapted pupil behaviour questionnaire (PBQ)

The PBQ was developed for and used extensively in school effectiveness studies, and is based on findings of the Elton Report [21]. It measures the types of classroom-based disruptive behaviours of particular concern to school staff. Teachers will complete the PBQ for all children in their class. The adapted version contains six items scoring 0 = never, 1 = occasionally, 2 = frequently. Items are summed with a higher total score indicting more disruptive behaviour.

#### *Teacher completed measures on themselves*

##### Teachers’ sense of efficacy scale [22]

A 12 item measure assesses the teacher’s perception of their sense of effectiveness as a teacher on three subscales (each with 4 items): *Student Engagement,Instructional Practice* and *Classroom Management*. Response is on a nine point scale for each item with anchors at 1 = nothing, 3 = very little, 5 = some influence, 7 = quite a bit and 9 = a great deal. Mean scores with a range of 4–36 are calculated for each scale with a higher score indicating a greater sense of efficacy.

##### Maslach burnout inventory- general survey [23]

A 16 item measure assesses aspects of ‘burnout syndrome’ which are recorded on three separate subscales: *Exhaustion, Cynicism* and *Professional Efficacy*. Respondents choose from seven options ranging from 0 = never, 1 = sporadic, 2 = now and then, 3 = regular, 4 = often, 5 = very often, 6 = daily. Mean scores are calculated for each subscale. A high degree of burnout is reflected in high scores on Exhaustion and Cynicism and low scores on Professional Efficacy.

##### Everyday feeling questionnaire (EFQ) [24]

A 10 item measure which records well-being over the previous four weeks. Half of the items focus on well-being and half on distress. Items are scored 0–4 for items with distress content and 4–0 for items with wellbeing content, with a maximum score of 40, with a higher score indicating increased distress.

#### *Child completed measures*

##### How I feel about my school [25]

Our group has developed and tested a measure of children’s attitude towards school. We recruited 268 pupils aged 4–7 years from three schools, who completed the seven-item *How IFeel AboutMy School*[25] questionnaire on two occasions, two weeks apart. Internal consistency was satisfactory (Cronbachs alpha =0.62 at Time 1, 0.67 at Time 2), with good test-retest reliability (intraclass correlation coefficient (ICC) = 0.63), and there were small but statistically significant correlations with parental reports on a parallel measure (Pearson correlation coefficient = 0.22 at Time 1 and 0.20 at Time 2). Children select one of the following responses for each item: sad (0), OK [1], happy [2], with a higher score indicating greater happiness at school. The potential range of the total score is 0–14 with a higher score indicating great enjoyment of school.

#### *Parent completed measures*

##### Strengths and difficulties questionnaire (SDQ [18])

Parents will also complete the parent rated version of the SDQ about their child at the four time points.

#### *Observer completed measures*

A random sample of schools will be chosen to complete these measures in order to validate the findings using the briefer questionnaire measures; they are not primary or secondary outcome measures for the trial. However, there will be some practical considerations that will restrain which schools we can enter for observations; some schools may refuse, and others may not have additional rooms available for the individual child assessments to take place. We will compare the schools that we visit with those that we cannot in detail to search for any potential biases, but as we are comparing one source of data with another on the same children, we do not anticipate that selection bias will have a major influence on our results. The observations will be completed by researchers who are independent to the core research team.

##### Wechsler individual achievement test (WIAT II-UK) [20]

The WIAT-II (Wechsler Individual Achievement Test – Second edition) is a psychometric assessment which measures reading, numerical attainment and language attainment in children from the age of 4. It is a psychometric assessment that is administered individually; it takes between 60–90 minutes depending on the child’s age and ability. It allows an assessment of the child’s functioning in these areas to be compared to national norms to determine the child’s achievement and ability in relation to other children their age.’ We will use the WIAT data to supplement the data gathered by the APP among a sub-sample of 50 children. Sub-tests of the WIAT II have been chosen to map onto the APP [18] for reading, spelling and maths and include Word reading, Reading comprehension and Spelling for literacy and numerical operations and mathematical reasoning for numeracy.

##### Teacher-pupil observation tool (T-POT [26])

Teacher-child interactions will be directly observed in a sub-sample of 20 classrooms (25% of teachers) using the TPOT. This is a structured real-time frequency count of defined teacher behaviours and types of teacher-child interaction that will be carried out by observers blind to allocation. Inter-rater reliability with two or three observers rating 21 primary school teachers was high (ICC = 0.78) [25]. The focus of the observation is the class teacher.

The T-POT uses continuous coding to look for nine different teacher behaviours and seven different behaviours from the children in the class. It measures behaviours that the TCM intervention specifically targets for change and therefore will be able to assess whether the teachers’ and children’s behaviour changes between T0 and T1. ‘*Teacher negatives*’ include: physical behaviours such as restraining/moving the child; verbal behaviours such as reprimanding the child; and not being explicit to a child about the behaviour that is expected. TCM aims to provide teachers with strategies to enable them to use more positive approaches and therefore reduce the need for these negative behaviours/verbalisations. This therefore will be measurable pre- and post-intervention at T0 and T1. The T-POT also looks at ‘ *teacher positives*’, which include praise, positive physical contact, positive facial expressions and verbalisations (e.g. laughing).

All teachers have their own unique style and therefore there are no ‘cut-off’ points to indicate good or bad practice, instead the T-POT encourages comparing change between two observations, particularly in relation to ratios of positive to negative behaviours. Scores on the T-POT will be compared to the relevant items on the classroom management and instructional practice subscales of the Teacher sense of efficacy scale, and the teachers report of the TCM strategies adopted at the end of the course for those in the intervention arm.

T-POT can also assesses a range of ‘*child positives’* and *‘child negatives’* to assess whether there is any potential impact of TCM on child behaviour in the classroom, but in STARS, due to time and financial restraints, the focus of observations is on the teacher behaviour.

### Economic resource-use data

#### *Parent completed measures*

##### Child and adolescent service Use schedule (CA-SUS) [27-29]

Resource-use information will be collected using the Child and Adolescent Service Use Schedule (CA-SUS), developed by one applicant (SB) in previous economic evaluations involving child and adolescent mental health populations [27-29]. Two versions of the CA-SUS will be used. Firstly, a brief self-report version to collect data on a limited set of key resource items (high cost and/or high use) from all parents at all four time points. Second, the full standard interview version of the CA-SUS will be used with a random sample of 50 parents in interview at T2 and T3 in order to validate and supplement the briefer self-complete version at all time points.

#### *Data from educational records*

Parent data on service use will be supplemented with data on educational service use at pupil level collected from schools.

### Process evaluation data

The process evaluation will include quantitative and qualitative methodologies; data collection and analysis will run parallel to the pilot and main phases.

#### *Quantitative data*

Data will be routinely collected relating to the administration of the TCM course. Group leaders complete standard checklists after each session that indicate which parts of the expected curriculum were covered. Standardised session evaluations and self-monitoring checklists are completed by teachers after each session to assist group leaders in planning, with a satisfaction questionnaire after the final session that collects data on the teachers’ application of the techniques covered in the course. TCM sessions will be filmed for supervision with the TCM programme developers, which allows the research team to analyse the videos for fidelity to model and contextual factors in each group. There will be eight TCM groups in total, each of 10 teachers, by the end of the trial with an additional four groups of 10 teachers from the two feasibility studies. This routinely collected data will be supplemented with data on recruitment, attendance and engagement with TCM, and with the qualitative data to provide contextual information on which to base recommendations about how TCM should be implemented successfully.

#### *Qualitative data*

Qualitative data will be collected using focus groups and semi-structured telephone interviews at different times throughout the study.

Focus groups will be used to collect data on the learning, uptake and use of TCM techniques in the classroom and informal transference to other staff members. In addition, a follow-up focus group with teachers, one year after completion of the TCM course will explore the maintenance of TCM techniques. We will undertake telephone interviews with Head teachers and SENCos in the second and third year of participation to collect data about differential use of support services, attribution to the teacher being TCM trained and perception of the place of TCM among other available sources of support.

Topic guides will be developed for both the focus groups and semi-structured (telephone) interviews. Interviews will be audio-recorded and transcribed.

Should time permit, we will undertake exploratory semi-structured interviews and/or focus groups with parents to explore their hopes and priorities for change in relation to their views and experiences of teacher classroom behaviour management and the promotion of mental health and well-being at school. This will, however, comprise a separate study with its own protocol and is mentioned in this protocol only because we will ask parents to indicate if they would be interested in participating as part of the feasibility work for the additional study.

### Other data

#### *Parent reported*

Parents will provide basic socio-demographic information about themselves and their child at baseline, and will include the following demographic details: child’s eligibility for free school meals, post code to link to the index of multiple deprivation, the number of children living in the household, housing tenure (rented or not), and the highest level of qualification of the parent(s) or carer(s).

#### *School reported*

We will gather school level data on the percentage of children eligible for school meals at recruitment and the index of multiple deprivation at lower super output area as a proxy for the school catchment area according to the school’s postcode [30]. We will also obtain information from schools about the type and level of emotional enrichment programmes (e.g. Socio-emotional aspects of learning, Thrive) being delivered in school, and how much other outside behavioural support they receive.

### Proposed sample size

#### *Randomised controlled trial*

Forty schools (clusters) will be randomised to each of the intervention and control arms, using one class from each school. Assuming that each class contains 30 pupils and that the recruitment rate is 70% (achieved among parents in the Helping Children Achieve trial [31] using the SDQ) we anticipate that 21 (i.e. 30*0.7) children from each class and a total of 840 (i.e. 21*40) children in each trial arm will participate in the study. Assuming 10% attrition for the children, we expect 19 of them to be followed-up at T3 in each class: a total of 760 (i.e. 19*40) children followed-up at T3 in each trial arm. As clusters are randomised the sample size calculation takes account of the correlation between participants’ responses within clusters. The intra-cluster correlation coefficient (ICC) for the primary outcome measure (SDQ total difficulties score) was estimated to be 0.15 using data from [Sayal *et al *32]. Using the formula VIF = 1 + (*n* - 1)* *ICC* presented in [Donner and Klar 33], the variance inflation factor (*VIF*) is 3.7 (i.e. 1 + [1]*0.15). The study will therefore be equivalent to a trial in which 205 (=760/3.7) participating pupils were individually randomised and provides 85% power at the 5% level of significance to detect a difference in the mean SDQ score between trial arms equivalent to an effect size of 0.3 of a standard deviation or a difference of 2 points on the raw SDQ scale. This would reduce the percentage of children classified in the borderline/abnormal range from 20% to 14% (http://www.sdqinfo.org/UKNorm.html) where borderline/abnormal is defined as those scoring 12 and above out of 40. Data from Goodman & Goodman [34] suggest that the odds of psychiatric disorder decrease by 33% for each 2 point decrease in the teacher SDQ and by 40% for each 2 point decrease in the parent SDQ.

#### *Nested qualitative study within process evaluation*

Sampling within the process evaluation will be purposive [35,36] to facilitate data collection of the views and experiences of participants, who can comment on the delivery, uptake and use of TCM strategies, appropriate to each phase of the trial. All intervention teachers in the trial will be invited to take part in focus groups. Sampling of Head-teachers and SENCos will reflect the aims of each trial phase.

##### Course experience and research processes

The aims of the process evaluation for the first cohort are to elicit a fuller understanding of the experiences of the course, course delivery and research process. All intervention group teachers (n = 10) will be invited to join a focus group after the course finishes. All head teachers from intervention group schools will be invited to take part in a telephone interviews (n = 10). Head teachers from control group schools (n = 5) will also be invited to take part in a telephone interview to elicit their views on the research processes.

##### Teacher learning and use of TCM strategies

In cohorts 2 and 3, all teachers in the intervention groups (15 in each year) will be invited to join a focus group aiming to elicit views and experiences of the learning, uptake and use of TCM strategies in the classroom. For teachers in their follow up year (i.e. second and third years of participation) all teachers from the previous year’s course will be invited to join a focus group to elicit views on maintenance of the use of TCM techniques in the classroom.

##### Impacts of course

In each of second and third years of participation, we plan to conduct interviews with up to 15 head teachers and 15 SENCos from the intervention schools In this phase we will aim to achieve a diversity of head-teachers and SENCos from a range of schools [36].

Although not the only issue affecting sample size in qualitative research, a guiding principle includes the concept of saturation [37,38]. Our sample size takes account of current guidance on optimising sample size in qualitative research.

### Statistical analysis

#### *Analysis of effectiveness*

All comparisons between trial arms will use the intention to treat principle where schools and participating pupils are analysed according to the arm to which they were randomised. Random effects linear regression models [39] will be fitted to compare means for continuous outcomes (including the primary outcome or SDQ total difficulties score) between the trial arms allowing for the correlation between outcomes of children from the same school specifying school effects as random. The method of marginal models using Generalised Estimating Equations with information sandwich (“robust”) estimates of variance and assuming an exchangeable correlation structure within school clusters [40] will be used to compare binary outcomes (e.g. borderline/abnormal versus normal status on the SDQ) between the trial arms, also allowing for clustering. A test of interaction will be implemented for each outcome to investigate whether the intervention effect differs across the three time points (T1, T2, T3).

Unadjusted analyses and analyses adjusted for important prognostic factors at the pupil level (e.g. child gender, year group and baseline SDQ score), cohort of recruitment and the school level (level of deprivation, urban versus rural status and whether involved in other emotional enrichment programmes) will be implemented. In a secondary analysis, interaction terms will be included to investigate possible differences in intervention effect (on the primary outcome SDQ score only) between pre-defined subgroups based on school and individual deprivation, low versus high baseline SDQ scores, length of teacher experience and year group. These sub-group analyses have been selected for a number of reasons. First, children experiencing socio-economic deprivation may benefit more than their more privileged peers. Second, previous work suggests that children with higher levels of difficulties experience the most benefit [10]. Third, teachers in our Feasibility Study suggest that newly-qualified teachers would gain the most from the course, and finally, there is a common belief that these interventions will have the biggest impact on younger children. The latter belief has focused research on very young children and this would be the only study to investigate TCM in children aged over seven years. P-values of 0.01 and less will be interpreted as providing evidence for interaction effects. Although the power to detect moderate subgroup interactions will be low, we are primarily interested in investigating the possibility of large quantitative interactions and not qualitative interactions where the direction of intervention effect differs between sub-groups. Demographic and baseline characteristics at the school and pupil level will be summarised using means and standard deviations (or medians and inter-quartile ranges) for quantitative characteristics and percentages for categorical characteristics.

#### *Cost and cost effectiveness*

TCM costs will be calculated using a standard micro-costing (bottom-up) approach [41], and will be based on teacher and trainer salaries plus on-costs (employers national insurance and superannuation contributions) and appropriate capital, administrative and managerial overheads. Costs for NHS hospital contacts will be taken from NHS reference costs [42]. Nationally applicable unit costs will be applied to all community health and social care contacts [43], medications [44], crimes and criminal justice resources [45,46]. The costs of schooling and school based services will be taken from various sources including Ofsted reports (the UK inspectorate and regulatory body for schools in England; http://www.ofsted.gov.uk) and published documents [47,48].

Despite the often skewed nature of costs, mean costs will be compared using standard parametric tests and the robustness of the results confirmed using bootstrapping [49]. The advantage of this approach, as opposed to logarithmic transformation or non-parametric tests, is the ability to make inferences about the arithmetic mean, which is more meaningful from a budgetary perspective [50].

The primary economic evaluation will explore cost-effectiveness at the T3 follow-up. Cost-effectiveness will be measured initially in terms of the primary outcome measure (SDQ). Cost-effectiveness will be assessed using the net benefit approach [51]. Uncertainty around the cost and effectiveness estimates will be represented by cost-effectiveness acceptability curves [52,53]. A joint distribution of incremental mean costs and effects for the two groups will be generated using non-parametric bootstrapping to explore the probability that each of the treatments is the optimal choice, subject to a range of possible maximum values (ceiling ratio) that a decision-maker might be willing to pay for an additional unit of outcome gained. Cost-effectiveness acceptability curves will be presented by plotting these probabilities for a range of possible values of the ceiling ratio [54]. These curves are a recommended decision-making approach to dealing with the uncertainty that exists around the estimates of expected costs and expected effects associated with the interventions under investigation and uncertainty regarding the maximum cost-effectiveness ratio that a decision-maker would consider acceptable [53,55]. To explore the longer-term implications of TCM, data from the RCT will be extrapolated and supplemented with data from the literature using decision analytic modelling techniques [56], in line with methods used to model the long-term impacts of parenting interventions for the prevention of persistent conduct disorders in children [57].

#### *Qualitative analysis*

All audio-taped qualitative data will be transcribed verbatim and anonymised. Data will be stored using Nvivo software http://www.qsrinternational.com/products_nvivo.aspx and will be password protected. Analysis will be guided by a realist perspective, to identify experiences as the lived ‘reality’ of participants, but we are also interested in the ways in which participants account for their experiences within the context of the trial and their own schools based experiences [58]. Thematic analysis of interview and focus group data will be both theoretically driven by the research questions and allow for more inductive analysis whereby emergent themes are also identified. This mixed approach will help explicate patterns of experience and views of teachers, head-teachers and SENCos. As highlighted previously, analytical interests in the study vary across the different trial data collection periods. During the first year, analysis will focus on the research processes (teachers and head-teachers), while during the last two years, the focus will on the TCM intervention, use in the classroom including identification of key contexts, influences and transference (teachers, head-teachers and SENCos). Analytical focus within the year following TCM course completion will be on maintenance of TCM skills in the classroom and differentials in use of services.

In our analysis, ‘keyness’ of themes does not relate to incidence of occurrence but to whether a theme captures information relevant to the research questions, in this case relating to a range of trial processes [59]. The Framework Approach [36] will be used to manage data and aid systematic analysis (description and summary of key themes, patterns and links in the data), allowing the researcher to move between levels of abstraction during analysis and between a theory driven and more inductive approach, while also displaying the relevant data sources. This approach will help maintain a focus on the process evaluation objectives for the different phases of the study.

Summary and illustrative data will be available, relevant to the aims of the qualitative research for each phase, to facilitate further interpretation and discussion of which processes worked well or not so well within the main trial. A number of methods will be adopted to enhance rigour during analysis including: checks for thematic saturation and consistency [37]; shared analysis; analytical discussions will be recorded; self-reflective memos will also be kept by researchers [60,61] and we will undertake a deviant case analysis, which involves a re-interrogation of data searching for new themes not covered in the initial data analysis [61].

## Plan of investigation

### Recruitment

There are 67 primary schools in Plymouth, 31 in Torbay and 314 in Devon (total 412), which provide plenty of opportunity to recruit the 80 schools required for the trial. The recruitment of schools will be staggered over a three year period, with 15 schools recruited in the first cohort, 30 schools in the second cohort and 35 schools in the third cohort. A recruitment strategy will be devised, and recruitment targets and rates continually monitored to ensure adherence to the trial plan. The Devon, Plymouth and Torbay Associations of Primary Headteachers are supporting the study and are taking an active role in publicising the study to their members.

### Retention

Our calculations indicate that even if as many as six schools (clusters) from each arm withdrew from the study (i.e. we retained 34 in each arm) then the study would still have 80% power at the 5% level of significance to detect our specified effect of 0.3 standard deviations on the total SDQ difficulties score. In order to maximise retention, we will update participating schools, families and other key stakeholders about the study’s progress through termly newsletters as well as feeding into the newsletters of relevant organisations such as the Devon, Plymouth and Torbay Associations of Primary Head Teachers. Parents will be offered a voucher in recognition of their time spent on the completion of questionnaires and interviews and control teachers will be offered TCM if they provide data at T0 and T1. Teachers will also be offered incentives for questionnaire completion at each data collection point. As a result of feedback from our first Feasibility Study, we will recommend that head teachers do not nominate teachers who have additional management roles that may compete with TCM for time away from the classroom. The upper age limit of recruitment in Year 4 classes means that the follow-ups can be completed while even the older children remain at their primary school. In order to make their participation in the study as straightforward as possible and resolve any issues quickly, each school will have a named researcher to build a collaborative relationship.

### Data management

All confidential data will be held in accordance with the Data Protection Act. Each school and participant included in the trial will be assigned a unique identifier and all data will be stored without identifying details. Data will be held on a secure database on a password-protected computer at the Peninsula Medical School. Access to data will be restricted to the research team. All trial documentation will be retained for ten years. The Peninsula Clinical Trials Unit (PenCTU) will develop the study database in addition to carrying out randomisation. It is envisaged that the teachers will complete the questionnaire measures on themselves and the children using a web-based tool, which will also be designed and maintained by the PenCTU.

### Project timetable and milestones

The timing of recruitment, assessment of participants and delivery of the intervention is dictated by the school academic year between September and July. Milestones for the trial have been set according to this criteria and a rolling programme of recruitment will take place over three academic years. Three cohorts of schools will be recruited over the first three years of the study. Follow-up of these cohorts will overlap with recruitment and will take place between the second and fifth years of the study. Data entry and process evaluation will be ongoing, and economic evaluation and analysis will take place between the second and fifth years of the study. The trial data analysis and report writing will take place during the fifth year.

## Study management

STARS will be managed by a core research team who will meet weekly to review progress and manage the data collection and data entry (TF, VE and research workers). There will be three main management committees:

### Trial management group

The senior management team (TF, SL, SB, OU, SS, BN, VE) will meet on a bi-monthly basis to review progress and set targets.

### Trial steering committee (TSC)

The TSC will be chaired by Professor Paul Stallard, a clinical psychologist who has led several trials of interventions in schools, and will also include members of our User Advisory Group with representatives from specialist educational professionals, teachers and parents. The TSC will meet at the beginning of the trial and annually thereafter to oversee its conduct.

### Data monitoring and ethics committee (DMEC)

An independent DMEC will consist of an independent statistician (Chair), a mental health and/or educational practitioner and an academic researcher. The DMEC will meet annually and if necessary in response to any serious untoward incidents to ensure that recruitment and retention is sufficient for the trial to continue and to review the analytic plan. The DMEC will report to the TSC in the first instance.

The University of Exeter will act as the Sponsor for the study. STARS will be hosted in the Child Health Research Group, which has experience in the successful delivery of community based paediatric trials.

## User advisory group

User involvement has been essential at all stages of the design, planning and implementation of the STARS programme of work. A User Advisory Group (UAG) has been established comprising parents, teachers, head teachers, behavioural support staff and TCM group leaders. Formal meetings have been held during the design stage of the study with much additional informal contact between meetings providing an iterative process of study development. The UAG has provided essential advice on acceptability of the study to parents and teachers, as well as recruitment and data collection procedures.

## Ethical arrangements

This study has been reviewed and approved by the Ethics Committee of the Peninsula College of Medicine and Dentistry.

### Consent to participate

Obtaining consent for this trial will be a four stage process.

1) Head teachers: after receiving the information leaflet and having the opportunity to discuss the implications of the study, head teachers will consent for the school to participate in the trial and will be asked to nominate one teacher to attend the TCM course.

2) Teachers: the nominated teacher will be given an information leaflet and given the opportunity to discuss the implications for their own and their class’ participation. Teachers will make their own decision about whether to participate. A potential ethical issue could arise if a teacher feels coerced by the head teacher to attend the course or feels that nomination is a criticism of their practice. These issues will be explored in the qualitative aspect of the process evaluation. The teachers in our UAG reassure us that the situation is unlikely to arise as it would risk damaging the relationship between the head and their staff. Our information sheets and promotional newsletters are designed to emphasise that the course hones skills, allows times for reflection and has something to offer at all levels of experience to try and avoid teachers who are nominated to attend from feeling criticised.

3) Parents: an information leaflet about the trial will be sent via school to the parents of all children in that teacher’s class. This will explain that if parents want to opt their child out of the trial, they need to return a form by a specified date (two weeks later), otherwise consent will be inferred. Parents will be able to opt themselves and their child out of the measurements but not opt the teacher or school out of the study.

Written parental consent will be sought separately for the literacy/numeracy assessment observational measures (WIAT II [20]) and economic interviews (CA-SUS [27-29]).

 4) Children: if parents have not opted their child out of the trial, the child’s verbal assent will be obtained before they complete the questionnaire measure on each occasion. Should a child become distressed or appear reluctant during data collection, this will be assumed to indicate their wish not to complete the measure.

The nested qualitative study is likely to elicit sensitive and confidential data, and attention to ethics and participant confidence in and acceptance of researchers is crucial. Information about the process evaluation will be included in trial information sheets to teachers (focus groups) and headteachers and SENCos (telephone interviews). Written consent for participation in focus groups or individual telephone interviews will be sought at the same time as consent to take part in the trial. A reminder and additional information on focus groups will be given to teachers towards the end of the TCM course. Head teachers and SENCos will be sent a reminder and information about the telephone interview process in advance of the interviews. Consent will be checked verbally at the beginning of interviews and will be recorded.

### Assessment of harms and adverse effects

The main indicators of harm will be the questionnaires completed by the children, parents and teachers at all four time points. Questionnaires will be screened for signs of severe distress. This will be defined in relation to teachers as a report of the most negative views of the future and self, and being completely unable to enjoy life in the *Everyday Feeling Questionnaire*. A child will be defined as severely distressed if they report feeling sad in response to all seven questions of the *How I Feel About My School *[24] measure. Parent and Teacher SDQs at follow-up will be screened for reports of their child’s problems being “*much worse*”. Responses meeting the above criteria will be reported to TF or a nominated deputy on the same working day; TF is a clinical mental health practitioner and will contact the family, school or local safeguarding practitioners as necessary.

Any concerns detected in this way will be recorded on a standardised pro forma, a copy of which will be sent to the Data Monitoring and Ethics Committee (DMEC) who will assess how likely it is that the adverse event is related to the conduct of the trial or TCM and advise if they consider additional action should be taken. The same process will be activated in response to any concerns raised by participants at other times, either spontaneously or during the focus groups and interviews; the latter will actively seek evidence of adverse effects.

#### Supporting teachers

Teachers will be offered support that will be separate to the research and will include assistance in seeking appropriate help from their GP or occupational health should this seem necessary. We think it unlikely that any teachers will be significantly distressed for the following reasons: all measures are validated questionnaires that have been widely used in previous, much larger studies as well as in our Feasibility Studies, without reports of distress. In addition, the philosophy of the IY programmes is to create a supportive collaborative group dynamic that draws on the strengths of all participants with no one (including the group leaders) assuming the role of expert and feedback from teachers in our Feasibility Study was incredibly positive.

#### Supporting children

We will discuss children who seem distressed in terms of their questionnaire responses or verbal exchanges with the head teacher or nominated deputy, and will contact parents to explore their concerns and support them to access appropriate assistance. This procedure will be made explicit in all information sheets and explained verbally to children each time we collect data.

#### Safeguarding

There will be a clear safeguarding policy and procedure for the researchers to follow should any child protection concerns arise. All researchers have basic training about child safeguarding, and any concerns will be discussed within the same working day with TF, VE or a nominated deputy who will contact the head teacher and/or children’s services if appropriate. The type and duration of follow-up will be decided by the external agencies involved with supporting the child, parent or teacher as appropriate, with the full cooperation of the research team. All researchers in contact with children and schools will be have the necessary Criminal Records Bureau checks.

## Dissemination

### Publication plan

· STARS Feasibility study; British Journal of Education; submit Autumn 2012

· Reliability and Validity of How I Feel about My School: Target Journal - Child: Health Care and Development; submission planned for Autumn 2012

· The relationships between APP levels and standardised attainment tests; Target Journal - British Educational Research Journal; submission planned for October 2014

· Short-term (T1) Effectiveness and cost-effectiveness: Lancet submit October 2015

· Trajectory / Longer term effectiveness and cost-effectiveness (T2 and T3); Target Journal - The Lancet; submission planned for October 2017

· Programme to practice: A qualitative analysis of the experiences of and influences on teachers use of the Incredible Years Teacher Classroom Management Strategies. Target Journal - British Journal of Educational Research. Submission planned for 2015/16

· Combining methods in process evaluation of complex trials. A case-study of the Incredible Years Teacher Classroom Management Course. Target Journal - Evaluation. Submission planned for 2015

· Modelling of longer term economic impact into adulthood – BMC Public Health– Submission planned for December 2018

### Liaison with parents, practitioners and policy makers

Dissemination of our findings to all those working in relation to child mental health and education is essential. We have detailed plans for reaching the child mental health and the children and young people’s services communities and educational practitioners, academics and policy makers. Reports from all components of the project will be made available to NHS commissioning bodies and we will publish in peer reviewed academic journals to span both education and mental health publications as described above.

Key professional groups in relation to education include, but are not limited to, teachers (including head teachers, SENCo’s, learning mentors, behavioural support teachers and teaching assistants), educational psychologists and behavioural support teachers. In order to engage with these groups, we will offer presentations of our findings at key national and regional meetings, including those of the Association of Educational Psychologists and the British Psychological Society, the British Educational Research Association conference in the UK and the American Academy of Child and Adolescent Psychiatry conference in the US and the National Association of Special Educational Needs and the Social Emotional and Behavioural Difficulties Association conferences in the UK. In addition, we will seek to present through the national network of the Association for Child and Adolescent Mental Health, whose branches are multidisciplinary and include both health and education. We will offer presentations of the findings to voluntary agencies and support groups involved with child mental health, such as Young Minds, Action for Children, and the Mental Health Foundation.

We also plan to feed our findings back to the Teaching Agency within the Department for Education, which has a number of initiatives to improve the specialist training of teachers following the Lamb Enquiry Report [62]. One strand of these initiatives focuses on socio-emotional and behavioural difficulties. Continuing professional development may offer opportunities to engage with education-based practitioners. In most areas there are local academic networks for different educational professional groups, such as special educational needs coordinators, lead behaviour professionals and head teachers that link with regional and national networks and would allow further dissemination of information through meetings, bulletin boards and newsletters. Should the trial lead to clear recommendations that this training should be provided to teachers, we will seek to link with the head of the Schools Inspection Service in order to explore how our findings could lead to the incorporation of appropriate standards into the inspection regime.

## Discussion

This study will provide important information about whether the Teacher Classroom Management course influences child and teacher mental health and well-being in both the short and long term. It will also provide valuable insights into factors that may facilitate or impede any impact.

## Abbreviations

TCM, Teacher Classroom Management; SDQ, Strengths and Difficulties Questionnaire; RCT, Randomised Controlled Trial; APP, Assessment of Pupil Progress; IY, Incredible Years; TAU, Teaching As Usual; T0 T1, T2, T3, Time points for data entry corresponding to Time 0 (baseline) Time 1 (9 months after baseline) Time 2 (18 months after baseline) and Time 3 (30 months after baseline); SENCo, Special Educational Needs Coordinators – senior teachers with a special remit to monitor and support children with special educational needs; PBQ, Pupil Behaviour Questionnaire; EFQ, Everyday Feelings Questionnaire; WIAT-II, Wechsler Individual Achievement Test – Second edition; TPOT, Teacher-Pupil Observation Tool; CASUS, Child and Adolescent Service Use Schedule; ICC, Intraclass Correlation Coefficient; UAG, User Advisory Group; TSC, Trial Steering Committee; DMEC, Data Monitoring and Ethics Committee.

## Competing interests

None of the authors have competing interests.

## Authors’ contributions

All authors have contributed substantially to the development of the protocol and have approved the final version of the manuscript. TF is the principal investigator and led the design of the study and the application for funding. She led the preparation of this manuscript for submission. VE is the Trial Manager and led the writing of the protocol. SS is the qualitative researcher who designed and will lead the qualitative aspects of the process evaluation. SB is the health economist that designed and will lead the economic evaluation. OU is the trial statistician and advised on the sample size, randomisation plan and the developed the analytic plan for the effectiveness of the intervention. SL provided methodological guidance and expertise throughout the design of the study and the development of the protocol. All authors read and approved the final manuscript.

## Funder

This project is funded by the National Institute for Health Research Public Health Research programme (Project reference: 10/3006/07).

Professional Indemnity Insurance is provided to the University of Exeter with Markel (UK)Ltd policy no. SC1919X110VR/423.

Ethical approval was granted by the Peninsula College of Medicine and Dentistry Research Ethics Committee on 8/3/12 reference number 12/03/141.

## Sponsor

The University of Exeter will act as the Sponsor for the study. STARS will be hosted in the Child Health Research Group, which has experience in the successful delivery of community based paediatric trials.

There are no conflicts of interest for any of the researchers working on this study. The intervention will be delivered by group leaders contracted from the Learning and Development Partnership and Plymouth Parent Partnership, who will be independently contracted for this work. The group leaders will be supervised by Carolyn Webster Stratton and her team from the Incredible Years Foundation to ensure fidelity to model. Neither the group leaders nor the Incredible Years team will have any influence on the conduct of the research or the interpretation and dissemination of the results.

## Pre-publication history

The pre-publication history for this paper can be accessed here:

http://www.biomedcentral.com/1471-2458/12/719/prepub

## References

[B1] Collishaw S Maughan B Goodman R Pickles A Time trends in adolescent mental health J Child Psychol Psychiatry 2004 45 1350 1362 10.1111/j.1469-7610.2004.00335.x 15482496

[B2] Ford T Practitioner review: how can epidemiological surveys help us plan and deliver effective child and adolescent mental health services? J Child Psychol Psychiatry 2008 49 900 914 10.1111/j.1469-7610.2008.01927.x 18573144

[B3] Scott S Knapp M Henderson J Maughan B Financial costs of social exclusion: follow up study of antisocial children into adulthood BMJ 2001 323 191 195 10.1136/bmj.323.7306.191 11473907 PMC35269

[B4] Webster-Stratton C Reid JM Hammond M Preventing conduct problems; promoting social competence: a parent and teacher partnership in head start J Child Psychol Psychiatry 2001 30 283 302 10.1207/S15374424JCCP3003_2 11501247

[B5] Brouwers A Tomic W A longitudinal study of teacher burnout and perceive self-efficacy in classroom management Teach Teach Educ 2000 16 239 254 10.1016/S0742-051X(99)00057-8

[B6] Greene R Beszterczey S Katzenstein T Park K Goring J Are students with ADHD more stressful to teach? J Emot Behav Disord 2002 2 79 90

[B7] Weare K Nind M Identifying evidence-based work on mental health promotion in schools in Europe: an interim report on the DataPrev project Adv Sch Mental Health Promot 2010 3 37 45 10.1080/1754730X.2010.9715679

[B8] Whear R Thompson-Coon J Boddy K Ford T Racey D Stein K The effect of teacher-led interventions on social and emotional behaviour in primary school children: a systematic review Brit Educ Res Jin press.

[B9] Hutchings J Daley D Jones K Martin P Gwyn R Early results from developing and researching the Webster-Stratton incredible years teacher classroom management training programme in North West Wales J Children’s Services 2007 2 15 26

[B10] McGilloway S Mháille G Lodge A O’Neill D Kelly P Leckey Y Bywater T Comiskey C Donnelly M Positive classrooms: positive children; a randomised controlled trial to investigate the effectiveness of the incredible years teacher classroom management training programme in an Irish context (short term outcomes) 2011 Summary report [prepared for Archways accessed via http://www.incredibleyears.com on 12 April 2011].

[B11] Raver CC Jones SM Li-Grining CP Metzger M Champion KM Sardin L Improving classroom processes: preliminary findings from a randomized trial in Head Start settings Early Child Res Q 2008 23 10 26 10.1016/j.ecresq.2007.09.001 PMC2274905 18364994

[B12] Webster-Stratton C Reid J Stoolmiller M Preventing conduct problems and improving school readiness: evaluation of the incredible years teacher and child training programs in high risk schools J Child Psych Psychiatry 2008 49 471 488 10.1111/j.1469-7610.2007.01861.x PMC2735210 18221346

[B13] Baker-Henningham H Walker S Powell C Gardner MJ A pilot study of the incredible years teacher training programme and a curriculum unit on social and emotional skills in community pre-school in Jamaica Child Care Health Dev 2009 35 624 631 10.1111/j.1365-2214.2009.00964.x 19320645

[B14] The incredible years websitehttp://www.incredibleyears.com/ accessed 12 April 2011.

[B15] Patterson GR Coercive family process 1982 Castalia, Eugene, OR

[B16] Bandura A Social learning theory 1977 Prentice-Hall, Englewood Cliffs

[B17] Piaget J Inhelder B The psychology of the child 1962 Basic Books, New York

[B18] Goodman R Psychometric properties of the strengths and difficulties questionnaire (SDQ) J Am Acad Child Adolesc Psychiatry 2001 40 1337 1345 10.1097/00004583-200111000-00015 11699809

[B19] DCSF Getting to grips with assessing Pupils' progress (APP) in the primary phase 2009 DCSF, Nottingham

[B20] Wechsler D Wechsler individual achievement test-second UK edition (WIAT-II) 2005 Harcourt Assessment, London

[B21] Gray J Richer J Classroom responses to disruptive behaviour 1988 Macmillan Education,

[B22] Tschannen-Moran M Wollfolk H A Teacher efficacy; capturing an elusive construct Teach Teach Educ 2001 17 783 805 10.1016/S0742-051X(01)00036-1

[B23] Maslach C Jackson SE Leiter MP Maslach burnout inventory manual 1996 3 Consulting Psychologists Press, Palo Alto

[B24] Uher R Goodman R The everyday feeling questionnaire: the structure and validation of a measure of general psychological well-being and distress Soc Psychiatry Psychiatr Epidemiol 2009 45 3 413 423 19466369 10.1007/s00127-009-0074-9

[B25] Higginson H Chan Seem E How I feel about my school” (HIFAMS) questionnaire validation: a measure of happiness in primary school children. Final year dissertation submitted to the School of Psychology, University of Exeter as part of the requirements for a BSc in Psychology

[B26] Daley D Hutchings J Jones K Eames C Whitaker CJ The teacher–pupil observation tool (T-POT) development and testing of a New classroom observation measure School Psychol Int 2010 1 3 229 249

[B27] Byford S Harrington R Torgerson D Kerfoot M Dyer E Harrington V Woodham A Gill J McNiven F Cost-effectiveness analysis of a home-based social work intervention for children and adolescents who have deliberately poisoned themselves: the results of a randomised controlled trial Br J Psychiatry 1999 174 56 62 10.1192/bjp.174.1.56 10211152

[B28] Harrington R Peters S Green J Byford S Woods J Randomised comparison of the effectiveness and costs of community and hospital based mental health services for children with behavioural disorders BMJ 2000 321 1047 1050 10.1136/bmj.321.7268.1047 11053174 PMC27511

[B29] Barrett B Byford S Chitasbesan P Kenning C Mental health provision for young offenders: service use and cost Br J Psychiatry 2006 188 541 546 10.1192/bjp.bp.105.010108 16738344

[B30] Office of Deputy Prime Minister The English indices of deprivation: summary (revised) 2004 TSO, London

[B31] Beckett C Kallitsoglou A Doolan M Ford T Scott S National academy of parenting research 2010 2010 Institute of Psychiatry, Kings College London

[B32] Sayal K Hornsey H Warren S MacDiarmid F Taylor E Identification of attention deficit/ hyperactivity disorder: a school based intervention Soc Psychiatry Psychiatr Epidemiol 2009 41 806 813 16841146 10.1007/s00127-006-0100-0

[B33] Donner A Klar N Design and analysis of cluster randomization trials in health research 2000 Arnold, London

[B34] Goodman A Goodman R Strengths and difficulties questionnaire as a dimensional measure of child mental health J Am Acad Child Adolesc Psychiatry 2009 48 400 403 10.1097/CHI.0b013e3181985068 19242383

[B35] Humberman AM Miles MB The qualitative Researcher’s companion 2002 Sage, London

[B36] Richie J, Lewis J Qualitative research practice: a guide for social science students and researchers 2008 Sage, London

[B37] Mason M Sample size and saturation in PhD studies using qualitative interviews [63 paragraphs]. forum qualitative sozialforschung / forum Qualitative Social Research 2010 11 3 Art. 8 [http://nbn-resolving.de/urn:nbn:de:0114-fqs100387].

[B38] Guest G Bunce A Johnson L How many interviews are enough? an experiment with data saturation and variability Field Methods 2006 18 59 82 10.1177/1525822X05279903

[B39] Goldstein H Multilevel statistical models 2003 3 Hodder Arnold, London

[B40] Hanley JA Negassa A deB Edwardes MD Forrester JE Statistical analysis of correlated data using generalized estimating equations: an orientation Am J Epidemiol 2003 157 364 75 10.1093/aje/kwf215 12578807

[B41] Drummond M Sculpher MJ Torrance GW Methods for the economic evaluation of health care programmes 2005 OUP, Oxford

[B42] Department of Health NHS reference costs 2010 Department of Health, London

[B43] Curtis L Unit costs of health and social care 2010 Personal Social Services Research Unit, University of Canterbury at Kent

[B44] British Medical Association and the Royal Pharmaceutical Society British national formulary 2010 BMJ Group and Pharmaceutical Press, London

[B45] Dubourg R Hamed J The economic and social costs of crime against individuals and households 2003–4 2005 Home Office, London

[B46] HM Prison Service Prison service annual report and accounts 2010 The Stationery Office. London, London

[B47] Berridge D Beecham J Brodie I Cole T Daniels H Knapp M MacNeill V Costs and consequences of services for troubled adolescents: an exploratory analytic study 2002 Department of Health Report

[B48] Independent Schools Council Annual census 2005 Independent Schools Council

[B49] Efron B Tibshirani RJ An introduction to the bootstrap 1993 Chapman & Hall, New York

[B50] Barber JA Thompson SG Analysis of cost data in randomized trials: an application of the non-parametric bootstrap Stats Med 2000 19 3219 3236 10.1002/1097-0258(20001215)19:23<3219::AID-SIM623>3.0.CO;2-P 11113956

[B51] Stinnett AA Mullahy J Net health benefits: a new framework for the analysis of uncertainty in cost-effectiveness analysis Med Decis Making 1998 18 2S S65 S80 10.1177/0272989X98018002S09 9566468

[B52] Van Hout BA Al MJ Gilad S Gordon GS Rutten FH Costs, effects and C/E-ratios alongside a clinical trial Health Econ 1994 3 309 319 10.1002/hec.4730030505 7827647

[B53] Fenwick E Byford S A guide to cost-effectiveness acceptability curves Br J Psychiatry 2005 187 106 108 10.1192/bjp.187.2.106 16055820

[B54] Fenwick E Claxton K Sculpher M Representing uncertainty: the role of cost-effectiveness acceptability curves Health Econ 2001 10 779 87l 10.1002/hec.635 11747057

[B55] Claxton K The irrelevance of inference: a decision-making approach to the stochastic evaluation of health care technologies J Health Econ 1999 18 3 341 64 10.1016/S0167-6296(98)00039-3 10537899

[B56] Philips Z Ginnelly L Sculpher M Claxton K Golder S Riemsma R Woolacott N Glanville J Review of guidelines for good practice in decision-analytic modelling in health technology assessment Health Technol Assess 2004 8 36 10.3310/hta8360 15361314

[B57] Bonin EM Stevens M Beecham J Byford S Parsonage M Knapp M, McDaid D, Parsonage M Parenting interventions for the prevention of persistent conduct disorders Mental health promotion and mental illness prevention: the economic case 2011 Dept. Health, London

[B58] Greenhalgh T Russell J Why Do evaluations of eHealth programs fail? an alternative Set of guiding principles PLoS Med 2010 7 11 e10000360 10.1371/journal.pmed.1000360 PMC2970573 21072245

[B59] Braun V Clarke V Using thematic analysis in psychology Qual Res Psychol 2006 3 2 77 101 10.1191/1478088706qp063oa

[B60] Padgett DK Qualitative methods in social work research 2008 2 Sage, Los Angeles

[B61] Silverman D Interpreting qualitative data: methods for analysing talk, text and interaction 2003 Sage, London

[B62] Lamb R Lamb inquiry: special educational needs and parental confidence 2009 DCSF, Nottingham

